# Effectors of Pregorexia and Emesis among Pregnant Women: A Pilot Study

**DOI:** 10.3390/nu14245275

**Published:** 2022-12-10

**Authors:** Alexandros Gerontidis, Maria G. Grammatikopoulou, Christos Tzimos, Konstantinos Gkiouras, Eleftheria Taousani, Loukas Athanasiadis, Dimitrios G. Goulis

**Affiliations:** 1 Department of Medicine, Faculty of Health Sciences, Aristotle University of Thessaloniki, GR-56429 Thessaloniki, Greece; 2 Department of Rheumatology and Clinical Immunology, Faculty of Medicine, School of Health Sciences, University of Thessaly, Biopolis, GR-41110 Larissa, Greece; 3 Northern Greece Statistics Directorate, Hellenic Statistical Authority, 218 Delfon Str., GR-54646 Thessaloniki, Greece; 4 Department of Midwifery, Faculty of Health Sciences, International Hellenic University, Alexander Campus, GR-57400 Thessaloniki, Greece; 5 Unit of Reproductive Endocrinology, 1st Department of Obstetrics and Gynecology, Medical School, Faculty of Health Sciences, Aristotle University of Thessaloniki, 76 Agiou Pavlou Street, GR-54629 Thessaloniki, Greece; 6 3rd Department of Psychiatry, Medical School, Aristotle University of Thessaloniki, 76 Agiou Pavlou Street, GR-54629 Thessaloniki, Greece

**Keywords:** orthorexia, disordered eating, pica, emesis, hyperemesis, nausea, eating disorder, gluten-free diet, vegetarianism, herbal medicine, maternal obesity

## Abstract

During pregnancy, women tend to improve their lifestyle habits and refine their dietary intake. Quite often, however, these dietary improvements take an unhealthy turn, with orthorexia nervosa (ON) practices being apparent. The aim of the present pilot cross-sectional study was to assess the prevalence of ON tendencies and the incidence of pica and record diet practices in a sample of pregnant women. A total of 157 pregnant women were recruited through private practice gynecologists during the first months of 2021. Nutrition-related practices were recorded, orthorexic tendencies were assessed using the translated and culturally adapted Greek version of the ORTO-15 questionnaire, pica practices were evaluated with a binary question and nausea and emesis during pregnancy (NVP) was evaluated using the translated modified Pregnancy—Unique Quantification of Emesis and Nausea (mPUQE). Only two women reported pica tendencies, with ice and snow being the consumed items. The majority (61.1%) of women reported improving their diet since conception was achieved. Folic acid and iron oral nutrient supplements (ONS) were reportedly consumed by the majority of participants (87.9% and 72.6%, respectively) and 9.6% reported using herbal medicine products. The ORTO-15 score was reduced with tertiary education attainment, ART conception, being in the third trimester of pregnancy, consumption of folic acid and MV supplements and was only increased among women who were at their first pregnancy. The majority of participants experienced severe NVP and the remaining experienced moderate NVP. NVP was associated with lower hemoglobin levels, lack of supplementary iron intake, avoidance of gluten-containing foods, as well as with increased gestational weight gain. The results highlight the need to screen pregnant women for disturbed eating behaviors and nutrition-related problems, in order to ensure a healthy pregnancy outcome.

## 1. Introduction

Pregnancy is a critical period in a woman’s life, where attaining good health is of outmost importance for both the mother and the fetus. During this time, mothers-to-be are inclined to improve their lifestyle habits by quitting smoking [1] and refining their dietary intake [2,3,4]. The notable improvement in lifestyle behavior taking place during gestation is, in fact, explained by the psychological theory [5]. Gestational maternal behavioral change is propelled by the motivation to improve lifestyle that is innately driven in the mothers and facilitated by community expectations, and additionally triggered by the development of the close maternal–fetal bond [6].

It has been suggested, however, that this motivation and effort to improve health and pregnancy outcomes can often reach unhealthy levels [7]. With obvious and salient changes in body shape taking place, alongside modifications in eating patterns, the gestational period often triggers, or aggravates symptoms of disordered eating [7]. Subsequently, the prevalence of eating disorders (EDs) during pregnancy has been estimated to reach 7.5% [8], although empirical evidence indicates that atypical EDs, including other specified feeding and EDs (OSFEDs) and unspecified feeding and EDs (USFED), appear to be more prevalent, though currently under-researched in the scientific literature [9,10,11].

Pregnant women with EDs often experience a variety of comorbidities, including binge eating, depression and anxiety, and excessive concern regarding gestational weight gain (GWG) [9]. A trio of causes have been identified as trigger factors for the development of EDs, OSFEDs and USFED during pregnancy, namely, (i) greater appetite levels, (ii) the need to improve lifestyle, including the adoption of a healthier diet and (iii) the body dissatisfaction associated with GWG [12]. When encountered during pregnancy and depending on their severity, EDs are associated with a variety of adverse events, including hyperemesis, vomiting, anemia and bleeding, impaired hydration status, sharp fluid and electrolyte shifts and an overall reduction in plasma volume, all of which can multiply the risk of maternal, obstetrical and fetal complications [13,14].

According to research, high levels of information and motivation about adhering to healthy eating patterns often propel the development of orthorexic tendencies during pregnancy [15]. Orthorexia nervosa (ON) is an OSFED characterized by a strong, unhealthy preoccupation for healthy “clean” eating [16], associated with perfectionism [17] and body-image dissatisfaction [18], the adoption of restrictive diets and inadequate intake of several micronutrients, leading to malnutrition and excessive fatigue [19,20]. When encountered during pregnancy, it is often termed as “pregorexia”. Pregnant women in particular, are eager to improve their lifestyle in a pursuit of better maternal, obstetrical and fetal outcomes. Thus, they appear prone to the development of ON tendencies, while, on the other hand, healthcare professionals and gynecologists are often underinformed and confused regarding ON symptoms, signs and prevalence [21].

In parallel, pica, the persistent consumption of non-nutritive substances [22], is also encountered frequently in pregnancy [12]. Pica is a paraphrase of the word “magpie,” a bird known for its unusual eating behaviors, eating almost anything available [23]. In the Diagnostic and Statistical Manual of Mental Disorders, Fifth Edition (DSM-5) [24], it is classified as an USFED. Hippocrates was the first to describe pica in pregnancy as follows: “If a pregnant woman wants to eat earth or charcoal, and eats it, the infant will show signs of those things on its head” [25]. Apart from humans, however, pica practices are also widespread in the animal kingdom [26], suggesting an etiology-driven evolutionary adaptation, possibly stemming from nutritional causes. Proposed explanations for pica practices during gestation include the elevated maternal psychological stress, increased appetite and hunger, cultural expectations, dyspepsia, underlying micronutrient deficiencies—iron in particular—and protection against toxins and pathogens [27]. The prevalence of pica practices during pregnancy varies greatly in the literature, although the lack of a specific tool to diagnose the behavior is evident [12].

The aim of the present pilot cross-sectional study was to assess the prevalence of ON tendencies, the incidence of pica and record diet practices in a sample of pregnant women.

## 2. Materials and Methods

### 2.1. Population

A total of 157 adult pregnant women were recruited through private practice gynecologists during the first months of the year 2021. Inclusion criteria involved (1) adult women, (2) at clinical pregnancy, (3) communicating effortlessly in the Greek language without requiring translators, (4) with willingness to participate.

Exclusion criteria involved (1) adolescent pregnant women, (2) with chemical pregnancy not verified via ultrasound, (3) experiencing difficulties in understanding the Greek language, (4) not willing to participate in the study. No criterion was set regarding possible comorbidities of participants. Table 1 details the characteristics of the study’s sample.

### 2.2. Ethics

The study’s protocol was approved by the Ethics Committee, Aristotle University of Thessaloniki’s Medical School (approval ID 2.332/24-11-2020). Every woman was adequately informed of the study’s aim before providing informed consent and participating in the research.

### 2.3. Instruments

#### 2.3.1. Evaluation of ON

Orthorexic tendencies were assessed using the Greek version of the ORTO-15 questionnaire [28,29]. The tool consists of 15 questions recording the frequency of orthorexia-related behaviors, with possible answers provided in a Likert scale. Although previous research has used the total ORTO-15 score in a scale format with a cutoff for ON diagnosis, due to the lack of a tool based on the newly established ON criteria [20], the use of thresholds to diagnose ON has been deemed as “unsafe” [30]. Instead, results are presented in a scale format, as a continuous outcome describing the degree of ON tendencies, instead of utilizing a cutoff for diagnostic purposes [29].

#### 2.3.2. Evaluation of Nausea and Vomiting during Pregnancy (NVP)

The severity of nausea and vomiting during pregnancy (NVP) was identified in the sample using the modified Pregnancy—Unique Quantification of Emesis and Nausea (mPUQE) [31]. The mPUQE is comprised of three questions in total, evaluating the frequency of NVP from the beginning of the pregnancy. Possible scores range between 3–15. Scores exceeding 13 points are indicative of severe NVP; mild NVP is diagnosed in women with mPUQE < 6; and participants with a score between 7–12 are considered as experiencing moderate NVP [31].

The mPUQE was translated and culturally adapted in the Greek language with permission from the authors [31], using the four-step forward–backward process, proposed by Guillemin et al. [32]. The reliability of the instrument was assessed with the Cronbach α for ordered variables [33].

#### 2.3.3. Evaluation of Pica

Pica practices were evaluated using the following binary (yes/no) question: “Do you consume non-food items (sponge, rocks, ice, snow, earth, chalk, clothes, walls, etc.)?” In the case of a positive answer, the items consumed were also recorded.

#### 2.3.4. Other Measures

Additional questions regarding the pregnancy (trimester, method of conception, etc.), comorbidities of the participating women, socio-economic status (SES), level of educational attainment, family status and typical questions regarding the participants’ diet (intake of dietary supplements, etc.), were also recorded.

### 2.4. Statistical Analyses

Normality in the distribution of the variables was assessed through graphs and the Kolmogorov–Smirnoff test. Data are presented as means ± standard deviation (SD) (normally distributed data), or as medians and their respective inter-quartile ranges (IQR) for non-normally distributed data. Categorical values are presented as *n* and their respective %.

Univariate logistic regressions were performed to identify the association between ON tendencies and mPUQE (dependent variables) and each independent variable. For the multivariable (MV) models, only those variables with a *p*-value < 0.500 were included. Data were analyzed using the Jamovi project (version 1.2.27.0) [34] and PASW Statistics 21.0 (IBM SPSS Inc., Hong Kong).

## 3. Results

### 3.1. Nutrition Practices and Health Problems

Table 2 details the nutrition-related practices reported by the participants. Out of 157 women in total, only 2 women reported having pica tendencies, with ice and snow (whenever possible) being the consumed items.

The majority (61.1%) of women reported improving their diet since conception was achieved. Folic acid and iron oral nutrient supplements (ONS) were consumed by the vast majority of participants (87.9% and 72.6%, respectively). Most women were avoiding herbal medicine, herbal drinks and the intake of non-nutritive sweeteners (NNS, including stevia, aspartame, etc.) Only 3.2% of the mothers-to-be reported being vegetarians and 10.8% were avoiding gluten-containing products.

Severe NVP was diagnosed in 101 participants, with the remaining having moderate NVP. The Cronbach α coefficient for the mPUQE was moderate (0.587). Supplementary Figure S1 presents the translated mPUQE scale in the Greek language.

Apart from pregnancy-related health issues, 1.3% of the women were diagnosed with type 2 diabetes mellitus and an additional 1.3% had hypertensive disorders of pregnancy. Gestational diabetes mellitus (GDM) was apparent in 5.7% of the sample, 19.7% reported experiencing constipation during pregnancy, while 12.1%suffered from hemorrhoids.

### 3.2. Factors Associated with ON Tendencies

Table 3 presents the univariable and multivariable models explaining ORTO-15 in the sample. In the univariable model, increased orthorexic tendencies were associated with a lower educational level, first parity, natural conception, being in the initial stages of the pregnancy (first or second trimester), avoidance of gluten products, fish, mollusks and crustaceans and lack of ONS supplementation with folic acid or multivitamin products (Table 3). In the multivariable model, ON tendencies were reduced with tertiary education, assisted reproduction technology (ART) conception, being in the last gestational trimester, consuming folic acid and MV supplements. In the multivariable model, ON tendencies were only increased among women who were experiencing their first pregnancy.

### 3.3. Factors associated with NVP

Table 4 details the regression analysis with mPUQE as the independent variable. In the univariable model, only low hemoglobin levels were associated with NVP. On the other hand, in the multivariable model, greater NVP was explained by lower hemoglobin levels, lack of iron ONS intake, avoidance of gluten-containing foods, as well as increased GWG.

## 4. Discussion

The present study reveals that the majority of pregnant women reported improving their diet since conception was confirmed. In this manner, they often exhibit ON tendencies, with tertiary education, ART conception, being in the third trimester of pregnancy, consuming folic acid and MV supplements reducing ON tendencies and being pregnant for the first time increasing ON practices. As for pica, the prevalence among participants herein was very small. In parallel, the majority of pregnant women appear to experience severe NVP, which was associated with lower hemoglobin levels, lack of iron ONS intake, avoidance of food products containing gluten, as well as increased GWG.

Pregnancy is a critical and nutritionally demanding time period for women, where the health of the fetus and a favorable pregnancy outcome are of utmost importance. In an effort to ensure a healthy pregnancy, women tend to improve their diet quality [2,35,36] in parallel with their nutrient intake, via the consumption of ONS [3,37,38]. This phenomenon is due to the fact that many pregnancies are not planned in advance; thus, quite often, an effort to compensate for previously unhealthy eating habits emerges post-pregnancy confirmation [2]. Despite the observed improvements in dietary intake, however, most women still follow a diet of suboptimal quality [3,39,40,41]. In the present sample, the majority of pregnant women admitted to improving in their diet since conception was confirmed. In parallel, nearly all participants reported consuming ONS, and in particular folic acid and iron. This is in agreement with a previous study conducted in Greece, which also revealed that the majority of pregnant women consumed ONS [3]. Herein, most women were avoiding herbal medicine, herbal drinks and the intake of NNS, in accordance with the clinical practice guidelines for an optimum pregnancy [42]. Only 3.2% of the participants reported being vegetarians and 10.8% were avoiding gluten products. According to a recent systematic review, meat avoidance often falls within the disordered eating spectrum [43] and vegetarianism may be associated with ED pathology and affective status [44]. Similarly, the avoidance of gluten-containing foods in lack of celiac disease diagnosis is frequently an underlying ED in disguise (Avoidant/Restrictive Food Intake Disorder, ARFID) [45] and does not come without adverse events [46].

Nonetheless, the tendency to improve maternal diet during gestation often reaches unhealthy levels [12]. In the present sample, ON tendencies were apparent, and this behavior was heightened among women who were pregnant for the first time. On the other hand, tertiary education attainment, ART conception, being in the third trimester of pregnancy, consuming folic acid and MV supplements appeared to reduce ON tendencies. According to Zeeni [47], during pregnancy, social media use and increased dependence on technological devices are associated with body-image dissatisfaction and appearance comparison, which could, in turn, propel ON tendencies. In a Turkish sample of childbearing women [15], higher levels of nutritional information and motivation were related to an obsession for “healthy eating”, propelling ON tendencies. According to research, ON can be quite restrictive, with patients often omitting specific “unhealthy” food groups from their diets, leading to nutritional deficiencies and medical complications [48,49]. Research on EDs during pregnancy noted that women with previous history of EDs were more likely to be vegetarian and follow a diet of good quality [50]. Unfortunately, a limited number of studies have been conducted to date assessing the ON tendencies during gestation, suggesting that the overall prevalence is approximately 26.6% [15]. However, it appears that ON often goes underdiagnosed in pregnancy, with gynecologists failing to assess symptoms and signs. Furthermore, ON tendencies appear to continue even post-partum [51], in a possible attempt at timely reduction of GWG.

As for pica, a very small proportion of participants herein reported eating ice and snow. Pagophagia (ice and freezer frost) is a common non-food item consumed during pica [12]. Research indicates that the prevalence of gestational pica is greatly dependent on the region studied, with a relatively low prevalence among Western societies (8.3%) and a greater one among pregnant women residing in less industrialized countries [12,52,53,54]. According to a meta-analysis [55], a pooled estimate of 27.8% of pregnant women experience pica. This is because, in some parts of the world, it is culturally accepted to consume non-nutritive substances [56]. Depending on the consumed substance, pica may result in fetal toxicity, long-term neurological disabilities for the newborn [57,58] as well as in maternal and perinatal mortality [59]. Furthermore, research is unanimous on the association between pica and low iron stores, or iron-deficiency anemia (IDA) [27,60,61], although such data were not available herein and the number of participants with pica did not allow for further statistical comparisons. Nonetheless, pica usually manifests as gastrointestinal distress, abdominal pain, gastritis, esophagitis, nausea and IDA [60,61]. Intervention studies suggest that iron infusion received parenterally can acutely resolve all cravings for non-food items, suggesting that low iron levels might in fact, be the triggering factor behind these cravings [12,61].

With regard to NVP, in the present sample, it was associated with lower hemoglobin levels, lack of iron ONS intake, avoidance of foods containing gluten, as well as with increased GWG. Due to the cross-sectional nature of the study, however, we cannot conclude if the observed effects are coincidental or have a cause–effect relationship. Nevertheless, NVP is associated with an increased psychological burden and consists of an important effector of quality of life, particularly during the first trimester of pregnancy [62]. Previous research has associated NVP with younger maternal age [63], primigravidas, lower level of attained education, maternal obesity [64], multiple gestation [65], low income levels and part-time employment status [66]. Across the literature, increased GWG and obesity in particular appear to be unanimous findings associated with NVP severity [63,67]. Furthermore, changes in the levels of adipocytokines have also been related to the physiological changes associated with NVP [68]. With regard to dietary intake, the Norwegian Mother and Child Cohort Study [63] associated NVP severity with greater carbohydrate intake; thus, the recorded avoidance of gluten in the present study might in fact be the correcting regime to reduce NVP severity among participating women. In parallel, discontinuation of iron supplementation was shown to reduce NVP symptoms [69]. Thus, the observed low Fe ONS intake herein associated with NVP might in fact consist of the method used by pregnant women to tamper down NVP symptoms. Nausea and vomiting are often worse in pregnant women with elevated human chorionic gonadotropin (hCG) levels, as seen in multiple gestations [70], and a plethora of research items concluded on the existence of a link between changes in hCG concentrations and NVP [71,72,73]. Previous research [74] has suggested that during pregnancy, women with atypical EDs exhibit greater odds of NVP.

An additional interesting finding of the present study involves the intake of herbal medicine and herbal drinks by pregnant women. A total of 9.6% of participants reported consuming such supplements during gestation. Research conducted in other areas of the world provided greater estimates, with 52% of Australian [75] and of 57.8% of U.K. pregnant women [76] reportedly using herbal medicine products. It is believed that herbal medicine products are safer than conventional medicine, and in many cultures, they are traditionally used to treat pregnancy complications, including NVP [77,78]. Furthermore, as they are often considered as “natural” and “safe” alternatives to conventional medicine, their use is frequently not reported to gynecologists and healthcare professionals [77], who, for their part, often neglect to ask about the use of such substances. Although some herbal medicine products may be safe for the mothers, not all are considered to be safe for the fetuses [79], while various adverse events have been associated with their use [42,77,80,81]. The obvious lack of knowledge on potential toxicity, paired with the possibility of interaction with other, more conventional treatments, can be harmful for both the mother and the child [77]. In this manner, it becomes obvious that educating pregnant women on the use of herbal medicine is important, whereas, in parallel, recording the use of herbal medicine products is also a pivotal issue from the health professional’s perspective [42,77,81].

Methodological limitations of the present study include the rather small sample size and the cross-sectional design. Furthermore, due to the nature of the ORTO-15, we could not assess the prevalence of ON in the sample. Nonetheless, the recent publication of the consensus on ON definition [20] is expected to aid the development of ON-specific tools, based on the suggested ON symptomatology. With regard to the mPUQE, a moderate Cronbach α was calculated for the tool, as a possible result of the small number of participants who answered positively in the three mPUQE questions. Last, but not least, the lack of a tool to diagnose pica is apparent in the literature; thus, we opted for the use of a binary question, as seen in previous research.

Undeniably, the prevalence of EDs, OSFED and USFED in pregnancy is a broad topic of varying grades of severity, with potentially threatening consequences for the fetus, including high APGAR scores, a greater risk for intra-uterine growth retardation (IUGR) and smaller head circumference, microcephaly, low birth weight and perinatal mortality [12,82,83,84]. When EDs, OSFED and USFED are diagnosed throughout gestation, a multidisciplinary treatment approach must be offered, focusing on the intake of adequate energy and nutrients, achieving appropriate GWG and improving pregnancy outcomes [12,85].

## 5. Conclusions

Pregnancy consists of an opportunity for intervention, improving lifestyle, developing long-term healthy eating habits and accepting physiological changes in body image [85,86]. As a result, many pregnant women improve their diet compared to pre-conception, while many also resort to ONS in order to increase selected micronutrient intake. This lifestyle improvement effort, however, frequently takes an unhealthy turn, with ON practices being apparent. The observed changes in body weight during pregnancy appears to trigger subsequent changes in the attitudes and behaviors related to eating [87]. In parallel, other USFED, including pica, may also appear during the gestational period, warranting further attention. Although the requirement to screen pregnant women for the detection of disturbed eating behaviors is of great importance and is highlighted in the literature, it is rarely the case in everyday clinical practice [85]. Clinical algorithms for the management of EDs are required [12,88] to aid gynecologists and health professionals in identifying women at risk of developing EDs and ensuring an optimal pregnancy outcome.

## Figures and Tables

**Table 1:** Characteristics of the participating women (mean ± SD or *n* and *%*) (N = 157).

Age (years)	32.7 ± 5.8
BMI at the beginning of gestation (kg/m^2^)	23.8 ± 4.1
GWG (kg)	7.8 ± 6.2
MUAC (cm)	27.6 ± 5.9
Nationality (Greek/Other) (*n*, *%*)	154 (*98.1%*)/3 (*1.9%*)
Ethnic minority (Latina/Chinese/African American/Roma) (*n*, %)	3 (*1.9%)*/1 (*0.6%*)/2 (*1.3%*)/1 (0.6%)
Level of educational attainment (secondary/tertiary/postgraduate) (*n*, %)	13 (*19.7%*)/84 (*53.5%*)/42 (*26.8%*)
Annual income per capita ≤5000/5001–10,000/10,000–15,000/>15,000 (EUR)	30 (*19.1%*)/48 (*30.6%*)/28 (*17.8%*)/20 (*12.7%*)
Family status (single/married/in a relationship/divorced) (*n*, *%*)	4 (*2.5%*)/141 (*89.8%*)/10 (*6.4%*)/1 (*0.6%*)
Trimester of gestation (1st/2nd/3rd) (*n*, *%*)	15 (*9.6%*)/65 (*41.1%*)/77 (*49%*)
Natural conception/ART	148 (*94.3%*)/9 (*5.7%*)
Single/twin/triple pregnancy (*n*, *%*)	148 (*94.3%*)/8 (*5.1%*)/1 (*0.6%*)
Parity (1st/2nd/3rd/4th/5th) (*n*, *%*)	91 (*58%*)/42 (*26.8%*)/17 (*10.8%*)/3 (*1.9%*)/4 (*2.5%*)

ART—assisted reproduction technology; BMI—body mass index; GWG—gestational weight gain; MUAC—middle upper-arm circumference.

**Table 2:** Nutrition-related practices of pregnant women (N = 157).

Nutrition Practices Followed by the Participants	Yes	No
	*n* (*%*)	*n* (*%*)
Vegetarianism	5 (*3.2%)*	152 (*96.8%*)
Consumption of foods containing gluten	140 (*89.2%)*	17 (*10.8%*)
Consumption of eggs and by-products	146 (*93%*)	11 (*7%*)
Consumption of milk and dairy	150 (*95.5%*)	7 (*4.5%*)
Consumption of red meat	151 (*96.2%*)	6 (*3.8%*)
Consumption of fish, mollusks and crustaceans	127 (*80.9%*)	30 (*19.1%*)
Pica behavior	2 (*1.3%*)	155 (*98.7%*)
Intake of folic acid ONS	138 (*87.9%*)	19 (*12.1%*)
Intake of MV ONS	32 (*20.4%*)	125 (*79.6%*)
Intake of Fe ONS	114 (*72.6%*)	43 (*27.4%*)
Intake of vitamin D ONS	63 (*40.1%*)	94 (*59.9%*)
Improvement of diet since gestation was achieved	96 (*61.1%*)	61 (*38.9%*)
Intake of herbal medicine and herbal drinks	15 (*9.6%*)	142 (*90.4%*)
Intake of NNS	39 (*24.8%*)	118 (*75.2%*)

Fe—iron; MV—multivitamin; NNS—non-nutritive sweeteners; ONS—oral nutrient supplements.

**Table 3:** Univariable and multivariable logistic regression models explaining ORTO-15.

Variables	Univariable				Multivariable			
	β	*p*	95% CI		β	*p*	95% CI	
			Lower	Upper			Lower	Upper
Tertiary education	−1.255	0.032	−2.403	−0.107	−1.321	0.015	−2.377	−0.266
First parity	1.533	0.010	0.379	2.687	1.234	0.026	0.151	2.318
Number of embryos	−0.394	0.719	−2.553	1.765				
Hb levels	0.026	0.65	−0.087	0.139				
ART conception	−3.056	0.015	−5.508	−0.605	−2.59	0.028	−4.891	−0.288
Third trimester of gestation	−1.279	0.029	−2.423	−0.135	−1.344	0.013	−2.396	−0.292
mPUQE score	0.292	0.262	−0.222	0.806				
MUAC	0.002	0.575	−0.004	0.007				
Intake of products containing gluten	−1.761	0.062	−3.609	0.088	−0.80	0.376	−2.58	0.98
Intake of folic acid ONS	−2.670	0.003	−4.400	−0.940	−2.763	0.001	−4.368	−1.158
Intake of MV ONS	−1.630	0.025	−3.049	−0.211	−1.83	0.006	−3.129	−0.531
Intake of Fe ONS	−0.399	0.546	−1.700	0.903				
Intake of herbal medicine/drinks	−0.606	0.545	−2.580	1.368				
Vegetarianism	0.054	0.974	−3.255	3.363				

ART—assisted reproduction technology; CI: Confidence intervals; Hb: hemoglobin levels; MUAC—middle upper-arm circumference; MV—multivitamin; mPUQE—modified Pregnancy—Unique Quantification of Emesis and Nausea [31]; ONS—oral nutrient supplementation; ORTO-15—orthorexia nervosa 15-item questionnaire [28,29].

**Table 4:** Univariable and multivariable logistic regression models explaining mPUQE.

Variables	Univariate				Multivariable			
			95% CI				95% CI	
	β	*p*	Lower	Upper	β	*p*	Lower	Upper
Tertiary education	0.018	0.966	−0.791	0.826				
First Parity	0.211	0.535	−0.463	0.884				
Hb levels	−0.049	0.027	−0.093	−0.006	−0.035	0.096	−0.077	0.006
ART conception	0.146	0.808	−1.041	1.334				
Second trimester of gestation	0.643	0.261	−0.489	1.775	0.301	0.328	−0.309	0.912
Intake of products containing gluten	−0.749	0.116	−1.687	0.19	−0.877	0.039	−1.708	−0.046
Folic acid ONS	−0.053	0.912	−1.018	0.911				
Fe ONS	−0.67	0.119	−1.517	0.177	−0.709	0.071	−1.481	0.062
Intake of herbal medicine/drinks	0.487	0.342	−0.53	1.504	0.468	0.324	−0.471	1.408
Vegetarianism	0.39	0.551	−0.903	1.683				
Pica practices	−0.305	0.857	−3.676	3.066				
GWG	0.053	0.151	−0.02	0.126	0.057	0.023	0.008	0.105
Has improved diet during pregnancy	0.003	0.992	−0.576	0.581				

ART—assisted reproduction technology; CI—confidence intervals; Fe—iron; GWG—gestational weight gain; Hb—hemoglobin levels; mPUQE—modified Pregnancy—Unique Quantification of Emesis and Nausea [31]; ONS—oral nutrient supplementation.

## Data Availability

All data are available upon request to the corresponding authors.

## Supplementary Materials

The following supporting information can be downloaded at: https://www.mdpi.com/article/10.3390/nu14245275/s1, Figure S1: Translation of the mPUQE in the Greek language.
