# Supplementary material for: Effectors of Pregorexia and Emesis among Pregnant Women: A Pilot Study
Source: Nutrients. 2022 Dec 10;14(24):5275. doi: 10.3390/nu14245275 (PMC9785442; doi:10.3390/nu14245275)
Supplement: Supplementary file 1 [file nutrients-14-05275-s001.zip › nutrients-2035637-Figure S1.pdf]

Τροποποιημένος ποσοτικός δείκτης συμπτωμάτων ναυτίας και υπερέμεσης κύησης
Modified Pregnancy-Unique Quantification of Emesis and Nausea Index mPUQE

Στις παρακάτω ερωτήσεις κυκλώστε αυτή που σας αντιπροσωπεύει, από την έναρξη της κύησης:

1. Σε μία τυπική ημέρα, για πόσο χρονικό διάστημα συνολικά νιώθετε ναυτία ή αίσθημα δυσφορίας (ανακάτεμα) στο στομάχι;

καθόλου	≤ 1 ώρα	2–3 ώρες	4–6 ώρες	> 6 ώρες
(1)	(2)	(3)	(4)	(5)

2. Σε μία τυπική ημέρα, πόσες φορές κάνετε εμετό;

≥ 7 φορές	5–6 φορές	3–4 φορές	1–2 φορές	Δεν κάνω εμετό
(1)	(2)	(3)	(4)	(5)

3. Σε μία τυπική ημέρα, πόσο συχνά νιώθετε αναγούλα ή τάση προς έμετο, δίχως να αποβάλετε κάτι;

Καμία	1–2 φορές	3–4 φορές	5–6 φορές	≥ 7 φορές
(1)	(2)	(3)	(4)	(5)

Βαθμολογία (σύνολο βαθμολογίας ερωτήσεων 1–3):
- ≤ 6 : Ήπιου βαθμού ναυτία και υπερέμεση κύησης
- 7–12 : Μέτριου βαθμού ναυτία και υπερέμεση κύησης
- ≥ 13 : Σοβαρού βαθμού ναυτία και υπερέμεση κύησης

Original article: Lacasse, A., Rey, E., Ferreira, E., Morin, C., Bérard, A. Validity of a modified Pregnancy-Unique Quantification of Emesis and Nausea (PUQE) scoring index to assess severity of nausea and vomiting of pregnancy. *Am. J. Obstet. Gynecol.* **2008;** 198, e1–e71.

Translated version: Gerontidis, A., Grammatikopoulou, M.G., Tzimos, C., Gkiouras, K., Taousani, E., Athanasiadis, L., Goulis, D.G. Effectors of pregorexia and emesis among pregnant women: a pilot study. *Nutrients* **2022**; 14.
